# Cows, Pigs and People: Enhanced Intensity-Based Clustering of Isomorphous Multi- Crystal Datasets in the Presence of Subtle Variations

**DOI:** 10.1063/4.0000877

**Published:** 2025-10-27

**Authors:** Amy J Thompson, James Beilsten-Edmands, Cicely Tam, Juan Sanchez-Weatherby, James Sandy, Halina Mikolajek, Danny Axford, Sofia Jaho, Michael A Hough, Graeme Winter

**Affiliations:** 1 Diamond Light Source, Didcot, Oxfordshire, United Kingdom; 2 Research Complex at Harwell, Didcot, Oxfordshire, United Kingdom; 3 University of Birmingham, Edgbaston, Birmingham, United Kingdom; 4 NE-CAT APS, Lemond, IL, USA; 5 Cornell University, Ithaca, NY, USA

## Abstract

The high-throughput data collection capabilities of modern X-ray facilities are challenging data processing pipelines to keep pace, while also remaining accessible to non-expert users. Rigorous analysis of multi-crystal data is necessary to sort through the data deluge, and the problem of which datasets to merge becomes an interesting scientific question. Lattice non-isomorphism is a key issue which has been well addressed (and automated) though techniques such as unit cell clustering (Foadi *et al.*, 2013). The effective separation of structurally isomorphous datasets with subtle differences (such as bound ligands, conformational changes or amino acid mutations), is a more challenging problem but has the promise to separate meaningfully different structures from crystal populations. Previous work has used the hierarchical clustering analysis of pairwise correlation coefficients to address this question (Matsuura *et al.*, 2023), although the interpretation of the resulting dendrograms can be ambiguous and thus difficult to automate. Hierarchical clustering can also be heavily dependent on the choice of linkage method, and the analysis of pairwise correlation coefficients does not distinguish between random and systematic errors. Work by Diederichs (Diederichs, 2017), however, extends this intensity-based clustering method to distinguish the effects of random and systematic errors, providing a clearer separation of datasets. These methods have since been extended within dials.cosym and applied in xia2.multiplex, a pipeline for the automatic scaling and merging of multi-crystal data (Gildea *et al.*, 2022, Gildea & Winter, 2018). These intensity-based clustering methods, however, were still missing the key element of automatic selection of relevant subsets of data, which is critical for analysing high-throughput data collections. In this presentation, clustering methods in dials.cosym and xia2.multipex are explored, showing the improved and automated methods to provide unambiguous separation of crystals of bovine, porcine and human insulin which have isomorphous lattices (Figure 1). Weighting of pairwise correlation coefficients, and the implementation of spatial density-based clustering algorithms enable such separation and are now available within the DIALS framework.
